# An arthropod *cis-*regulatory element functioning in sensory organ precursor development dates back to the Cambrian

**DOI:** 10.1186/1741-7007-8-127

**Published:** 2010-09-24

**Authors:** Savita Ayyar, Barbara Negre, Pat Simpson, Angelika Stollewerk

**Affiliations:** 1 Department of Zoology, University of Cambridge, Downing Street, Cambridge CB2 3EJ, UK; 2 School of Biological and Chemical Sciences, Queen Mary University of London, Mile End Road, London E1 4NS, UK

## Abstract

**Background:**

An increasing number of publications demonstrate conservation of function of cis-regulatory elements without sequence similarity. In invertebrates such functional conservation has only been shown for closely related species. Here we demonstrate the existence of an ancient arthropod regulatory element that functions during the selection of neural precursors. The activity of genes of the *achaete-scute *(*ac-sc*) family endows cells with neural potential. An essential, conserved characteristic of proneural genes is their ability to restrict their own activity to single or a small number of progenitor cells from their initially broad domains of expression. This is achieved through a process called lateral inhibition. A regulatory element, the sensory organ precursor enhancer (SOPE), is required for this process. First identified in *Drosophila*, the SOPE contains discrete binding sites for four regulatory factors. The SOPE of the *Drosophila asense *gene is situated in the 5' UTR.

**Results:**

Through a manual comparison of consensus binding site sequences we have been able to identify a SOPE in UTR sequences of *asense*-like genes in species belonging to all four arthropod groups (Crustacea, Myriapoda, Chelicerata and Insecta). The SOPEs of the spider *Cupiennius salei *and the insect *Tribolium castaneum *are shown to be functional in transgenic *Drosophila*. This would place the origin of this regulatory sequence as far back as the last common ancestor of the Arthropoda, that is, in the Cambrian, 550 million years ago.

**Conclusions:**

The SOPE is not detectable by inter-specific sequence comparison, raising the possibility that other ancient regulatory modules in invertebrates might have escaped detection.

## Background

The initiation of development of the nervous system in vertebrates and higher invertebrates involves the activity of proneural genes that encode transcription factors of the basic helix-loop-helix (bHLH) class [1]. Their expression in the neuroectoderm endows cells with neural potential and also contributes to the specification of neuronal identity. Proneural genes are conserved throughout the animal kingdom and fall into two main classes: the *achaete-scute *(*ac-sc*) and *atonal *(*ato*) gene families. They are initially expressed during development in groups/domains of equivalent neuroectodermal cells. An essential, conserved characteristic of proneural genes is their ability to restrict their own activity to single or a small number of progenitor cells within these domains [1]. This is achieved through a process called lateral inhibition, mediated by Notch signaling [2]. The Notch ligand Delta is up-regulated by proneural proteins in future neural precursors and activates the Notch signaling cascade in neighboring cells, resulting in down-regulation of proneural gene expression [3,4]. Repression of proneural genes is mediated by the products of the Notch target genes *Hairy*/*Enhancer of split*. This ancient regulatory network was probably inherited from the earliest Metazoa [5].

Regulatory sequences involved in the restriction of proneural gene expression from proneural domains to selected neural precursors have mostly been studied in *Drosophila melanogaster*, in particular with respect to the *ac-sc *genes and their role in the development of sensory bristles of the adult peripheral nervous system. The *D. melanogaster ac-sc *gene complex (AS-C) comprises four genes, three of which are required for bristle development. *ac *and *sc *are expressed in discrete proneural clusters through the activity of a number of independently acting *cis-*regulatory modules that are scattered throughout the approximately 150 kb of the AS-C and respond to positional cues [6-9]. Subsequently, the expression of *ac *and *sc *refines to single sensory organ precursors (SOPs) where high levels of Ac/Sc activate the third gene, *asense *(*ase*), whose expression is limited to SOPs [10-13]. Lateral inhibition and SOP expression is mediated by a specific *cis-*regulatory element, the SOP enhancer (SOPE) [14]. The SOPE contains binding sites for a number of transcription factors. Auto-regulation in the SOP relies on E boxes, binding sites for Ac, Sc and Ase, which activate their own transcription [15]. The E boxes also mediate repression in cells not selected to be SOPs: products of the *Enhancer of split *(*E(spl)*) genes activated by Notch signaling associate with Ac-Sc, leading to transcriptional repression [16]. Binding sites for NF-κB proteins, α boxes, are present and also mediate both activation and repression [14,17]. It is likely that low levels of NF-κB and high levels of Ac-Sc activate, whereas high levels of NF-κB and low levels of Ac-Sc repress, the neural program [18]. In addition, the SOPEs contain AT-rich sequences, β boxes, of unknown function and N boxes that, in the case of the *ac-*SOPE, have been shown to bind the transcriptional repressor Hairy [14,15,19,20]. All three genes bear their own SOPE. That of *ac *is in the promoter close to the transcription start site and differs from the others in being devoid of α boxes (unpublished observations, P. Simpson) [15,19]. It drives expression of reporter genes first in proneural domains and then in SOPs [15,19]. The SOPE of *sc*, positioned 3 kb upstream of the transcriptional start site, and that of *ase*, positioned in the 5' UTR, drive expression of reporter genes exclusively in the SOP [13,14]. The SOPEs are strongly conserved in other Drosophilidae.

Proneural genes of both the *ac-sc *and *ato *classes have undergone independent duplication events in different taxa. The *ato *gene family is much expanded in vertebrates whereas duplication of *ac-sc *genes has taken place in different groups of arthropods [21-24]. Previous data from available insect genomes have shown that while *ac-sc *genes have undergone a number of duplication events, all species analyzed bear a single *ase *gene. Conservation of both specific amino acid sequences and the SOPE in the 5' UTR suggest that the insect *ase *genes are derived from a common ancestor [22]. Here we show that *achaete-scute homologue *(*ASH*) and *ase*-like genes are present in arthropods other than insects. We present evidence that gene duplications separating proneural from precursor-specific (*ase-*like) functions possibly occurred independently in different arthropod groups and that a SOPE in UTR sequences in *ase-*like genes of all groups has been inherited from an ancestral *ASH*/*ase *precursor gene in the last common ancestor of the Arthropoda.

## Results

### Conservation of coding sequences suggest duplication and subfunctionalization of an ancestral arthropod gene into proneural and *ase-*like functions

A highly conserved bHLH domain characterizes proteins encoded by the *ac-sc *gene family, but outside this domain conservation is very low. Recently, two conserved domains were identified that, in insects, enable a distinction to be made between *ASH *genes that are expressed in proneural domains, called henceforth proneural *ASH *genes, and the sensory organ precursor-specific *ase *genes [22]. Firstly, proteins encoded by *ASH *genes contain a 16 amino acid carboxy-terminal domain (PDDEELLDYISWWQQQ) that is characteristic of all insect ASH proteins but is less well conserved in Ase proteins (50% identity or less). Secondly, Ase proteins have a characteristic five amino acid motif (hydrophobic-Lys-polar-Glu-hydrophobic) that is absent in all proneural ASH proteins outside the Diptera. These motifs allowed a clear subdivision of the *ASH *and *ase *genes in different orders of insects, which is upheld by phylogenetic analysis [22,25]. A single *ase *gene (but a variable number of *ASH *genes) is present in each species analyzed.

**Figure 1:**
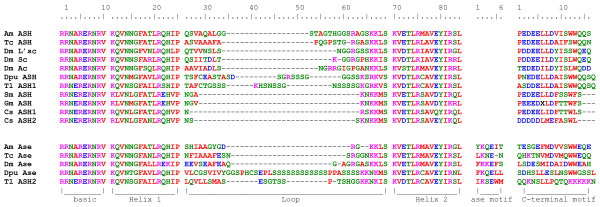
**Conserved domains of the arthropod ASH and Ase proteins**. Alignment of the bHLH domain, Ase motif and carboxy-terminal motif of ASH and Ase. Am, *Apis mellifera*; Cs, *Cupiennius salei*; Dm, *Drosophila melanogasster*; Dp, *Daphnia pulex*; Gm, *Glomeris marginata*; Sm, *Strigamia maritima*; Tc, *Tribolium castaneum*.

In order to classify proneural and precursor-specific genes in other arthropod groups, we applied the above criteria to recently published sequences. Two *ASH *genes were described in the crustacean *Triops longicaudatus *[24]. The authors show that the deduced amino acid sequence of Tl-ASH1 bears the ASH carboxy-terminal domain, while this sequence is not conserved in Tl-ASH2. We identified the Ase motif in Tl-ASH2, confirming that this gene is in fact an *asense *orthologue (Figure 1). Furthermore, we detected single *ASH *and *ase *orthologues, *Dpu-ASH *and *Dpu-ase*, in the *Daphnia pulex *genome (Daphnia Genome Consortium), which can clearly be distinguished by the presence of the respective domains (Figure 1). The spider *Cupiennius salei *(chelicerate) displays two *ASH *orthologues [23] but sequence analysis does not unambiguously distinguish a *bona fide ase *gene in this species. The carboxy-terminal domain of CsASH1 displays a greater similarity to that of insect and crustacean ASH proteins (56% amino acid identity) than does that of CsASH2 (30%). However, neither CsASH1 nor CsASH2 contain the five amino acid motif characteristic of Ase (Figure 1). A single orthologue has been identified in each of the myriapods *Glomeris marginata *[26] and *Strigamia maritima *(*Strigamia *Genome Project, Human Genome Sequencing Consortium, Baylor College). We are confident that there is only a single copy present in the *S. maritima *genome (see Materials and methods). They show 50% and 62% identity with the insect ASH carboxy-terminal domain, respectively, and 78% identity with the carboxy-terminal domain of CsASH1. They lack the Ase-specific motif and would appear to be *ASH *genes.

**Figure 2:**
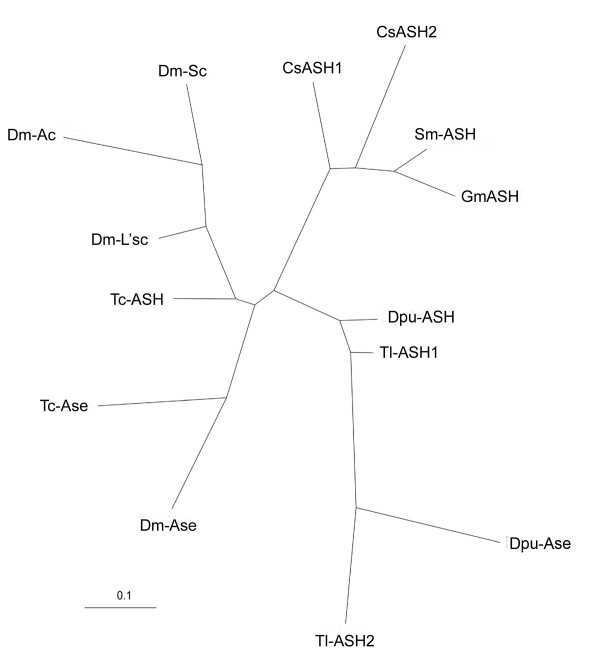
**Phylogenetic analysis of the arthropod ASH and Ase-like proteins**. ASH and Ase of insects group together, as do those of crustaceans, while the ASH proteins of myriapods and of chelicerates are arranged in a single group. Both the insect and the crustacean proteins are clearly subdivided into ASH and Ase groups. The spider proteins CsASH1 and CsASH2 are arranged in a group with the single myriapod orthologues. Cs, *Cupiennius salei*; Dm, *Drosophila melanogaster*; Dp, *Daphnia pulex*; Gm, *Glomeris marginata*; Sm, *Strigamia maritima*; Tc, *Tribolium castaneum*.

Earlier analyses suggested that the two *ASH *genes of *T. longicaudatus *and of *C. salei *arose from duplication events that are independent from those of insects and from each other [23,24]. We have performed a new phylogenetic analysis that includes the *D. pulex *sequences that were not previously available. All *ASH *and *ase *genes from the insects *D. melanogaster *and *Tribolium castaneum*, the crustaceans *T. longicaudatus *and *D. pulex*, the chelicerate *C. salei *and the myriapods *G. marginata *and *S. maritima *were included. The phylogenetic tree shows that the ASH and Ase proteins of insects group together, as do those of crustaceans, while the ASH proteins of myriapods and of chelicerates are arranged in a single group (Figure 2). Both the insect and the crustacean proteins are clearly subdivided into the ASH and Ase groups, that is, *T. castaneum *Ase groups with *D. melanogaster *Ase and *T. castaneum *ASH is arranged in a group with *D. melanogaster *Ac, Sc and the proneural protein Lethal of Scute (L'sc). Similarly, the *D. pulex *Dpu-Ase protein groups with *T. longicaudatus *Tl-ASH2, and Tl-ASH1 groups with Dpu-ASH, rather than with the insect orthologues (Figure 2). The spider CsASH1 and CsASH2 are arranged in a group with the single myriapod homologues (Figure 2). The analysis suggests independent duplication events in insects, crustaceans and chelicerates. However, two features confound the phylogenetic inference. First, the *ASH *and *ase *genes in the individual arthropod groups might have evolved at different rates - for example, a faster evolution of insect *ASH *and *ase *genes would prevent them from grouping with their crustacean orthologues. Second, the myriapod and the chelicerate *ASH *genes might group together because they have retained many ancestral homologies.

To gain further insight into patterns of gene duplication, we have directly tested three different tree topologies in support of: a single ancestral duplication giving an *ASH-*like and an *ase-*like gene at the base of the arthropods; independent duplications at the base of the insect-crustacean lineage and of the chelicerate-myriapod branch; and independent duplications at the base of each of the insect, crustacean and chelicerate lineages. The Shinodaira-Hasegawa test discards the first possibility. It supports a single duplication in the chelicerate-myriapod branch. However, it cannot distinguish whether a duplication took place in the last common ancestor of insects and crustaceans or whether it occurred independently in each of these groups. The presence of the Ase-specific domain (which was not used for construction of the phylogenetic tree), together with the position of the *ase*-SOPE (see below), favors a single duplication common to insects and crustaceans. Thus, within the limits of this analysis, which employs only very short sequences (66 amino acids), the data suggest an independent *ASH*/*ase-*like duplication in insects/crustaceans and chelicerates.

### *C. salei CsASH2 *rescues *ase-*specific defects in *D. melanogaster*

Insect AS-C genes are also distinguishable by their expression patterns: *ASH *genes are expressed in proneural domains prior to the segregation of neural precursors, in contrast to the *ase *genes, which are only expressed in neural precursors after they have been singled out [7,8,11,12,27,28]. In a similar fashion, the crustacean proneural gene *Tl-ASH1 *was shown to be expressed in clusters of cells, whereas *Tl-ASH2 *is expressed later in only a subset of the *Tl-ASH*-expressing cells [24], providing further evidence that *Tl-ASH2 *is likely to be an *ase *orthologue. Interestingly, in the spider, *CsAHS1 *is expressed in proneural domains whereas expression of *CsASH2 *is restricted to neural precursors (groups of precursors, instead of single cells, are formed in this species [23]). This suggests that *CsASH2 *might carry out an *ase-*like function. We therefore investigated whether *CsASH2 *can rescue the specific defects caused by a loss of *ase *activity in *D. melanogaster*.

**Figure 3:**
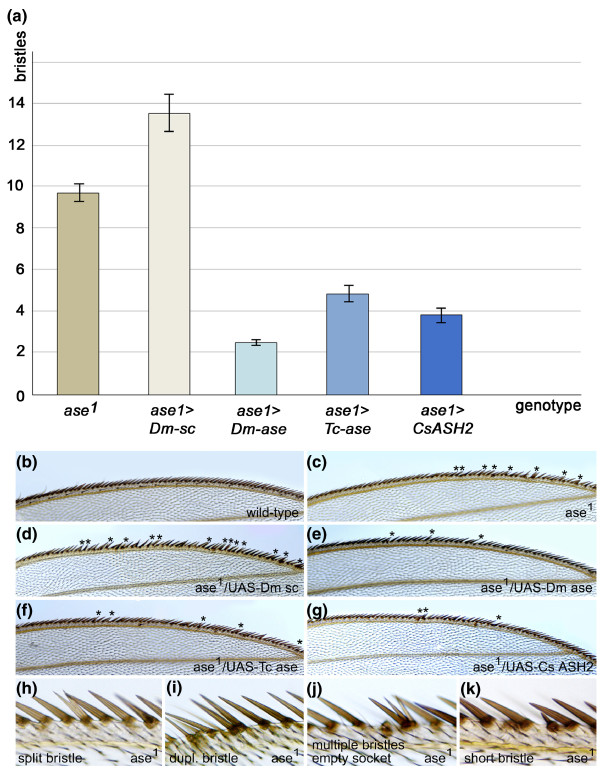
***C. salei CsASH2 *and *T. castaneum ase *can both rescue the bristle phenotype in *D. melanogaster ase^1 ^*mutants**. **(A) **Bar chart showing the extent to which *Dm-ase*, *Tc-ase *and *CsASH2 *can rescue the phenotype when ectopically expressed (*hsp70Gal4 > UAS Dm-sc/Dm-ase/Tc-ase*/*CsASH2*). In contrast, *Dm-sc *enhances the *ase^1 ^*phenotype (*hsp70Gal4 > UAS Dm-sc*). The number of bristles affected is given on the x-axis. Error bars indicate the standard error of the mean (see Additional file 1 for details). **(B-G) **Abnormally differentiated bristles (asterisks) on the anterior wing margin are shown for the genotypes indicated (genotypes as in (A)). The number of abnormal bristles is enhanced in (D) and reduced in (F, E, G). **(H-K) **Detail of the abnormalities at higher magnification.

Flies lacking *ase *function exhibit only a mild phenotype because activity of *ac*, *sc *and *senseless *compensates for most of the defects [11,13,29,30]. However, one defect is specific to *ase*: differentiation of the stout mechanosensory bristles of the triple row of bristles on the anterior wing margin is impaired [11,13]. In *ase^1 ^*mutant flies these bristles show variable defects that include a split shaft, two to three shafts arising from a single socket, an empty socket or a complete duplication (Figure 3H-K). When over-expressed, *Dm-ase*, but neither *Dm-ac *nor *Dm-sc*, has been shown to rescue these defects [11,13]. We found that *CsASH2*, as well as *Tc-ase*, display a rescuing activity comparable to that of *Dm-ase *(Figure 3A-G). The number of defective bristles in *ase^1 ^*flies is reduced from an average of 9.7 to 2.5, 4.9 and 3.9 in flies expressing *Dm-ase*, *Tc-ase *and *CsASH2*, respectively (Figure 3A-G; Additional file 1). *CsASH2 *can therefore substitute for functions specific to *Dm-ase*, which suggests that it might carry out precursor-specific *ase-*like functions in the spider.

### *ase-*like genes of insects, crustaceans and chelicerates and the *ASH *genes of myriapods bear a conserved regulatory sequence in the UTR

The *ase *gene of *D. melanogaster *bears a *cis-*regulatory sequence in the 5' UTR, the SOPE, that drives expression of a reporter gene in the SOP [13]. Although equivalent enhancer elements drive expression of *sc *and *ac *in SOPs, they are located upstream of the transcription start site [14,15,19]. Genome analysis indicates that the *ase *SOPE is conserved in the 5' UTR of the *ase *gene of other insects whereas no such sequence is found in the transcribed regions of insect proneural *ASH *genes [22]. The presence of a SOPE in the UTR therefore provides another feature with which to distinguish between *ASH *and *ase*-like genes.

**Figure 4:**
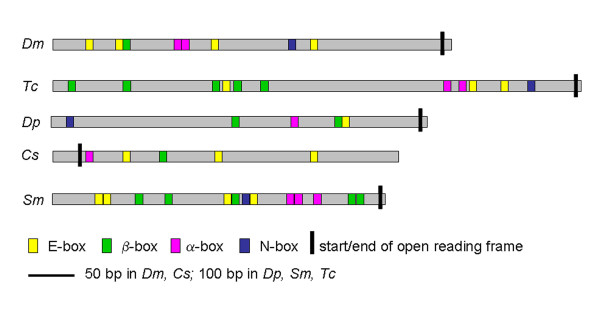
**Arrangement of transcription factor binding sites in the UTR of the arthropod *ase*-like genes**. Note that the SOPE covers a larger area in *D. pulex *(882 bp) and in *S. maritima *(1,052 bp) compared to *D. melanogaster *(297 bp), *T. castaneum *(247 bp) and *C. salei *(246 bp). See text for details. Yellow, E box; green, β box; pink, α box; blue, N box. *Dm*, *D. melanogaster*; *Tc*, *T. castaneum*; *Dp*, *D. pulex*; *Cs*, *C. salei*; *Sm*, *S. maritima*.

The SOPE bears binding sites for four specific transcription factors. Interestingly, we identified clusters of the relevant binding sites in the UTR of *D. pulex ase *and in *CsASH2 *of *C. salei*. The putative SOPE is located in the 5' UTR of *Dpu-ase *and in the 3' UTR of *CsASH2*. No such sequence is found in the UTR of *CsASH1*. Individual binding sites were identified by manual comparison of consensus sequences (Figure 4; Additional file 2). The *Dm-ase *SOPE, located 144 bp upstream of the start codon, contains four E boxes, two α boxes, one β box and one N box. We identified a putative SOPE in an additional insect, *T. castaneum*, which covers 1,145 bp of the 5' UTR starting 95 bp upstream of the *ase *open reading frame (ORF). It contains three E boxes, two α, five β and one N box (Figure 4; Additional file 2). In *D. pulex *the putative SOPE is located 1,048 bp upstream of the *ase *ORF and extends over 882 bp in the presumptive 5' UTR. One E box, one α box, two β boxes and one N box are present in this region (Figure 4; Additional file 2). The putative SOPE of *C. salei CsASH2 *is also close to the ORF but is located in the 3' UTR, between 3 bp and 249 bp downstream of the stop codon. It contains three E boxes, one α box, and one β box. No N box can be identified in this species (Figure 4; Additional file 2).

Remarkably, we also detected a putative SOPE in the 5' UTR of the single *S. maritima ASH *gene (*Strigamia *Genome Project, Human Genome Sequencing Consortium, Baylor College). It covers the region between 36 and 601 bp upstream of the ORF and contains four E boxes, three α boxes, five β boxes and one N box (Figure 4; Additional file 2). These data suggest that a *cis-*regulatory element located in the UTR, the SOPE, is conserved in the *ase-*like genes of insects, crustaceans and chelicerates and in the *ASH *gene of myriapods.

In order to identify conserved motifs and demonstrate the level of conservation, we generated sequence 'logos' [31] based on the aligned sequences of the individual arthropod SOPE boxes (Additional file 3). The α box shows the most degenerate consensus sequence, with conservation limited to the central part of the NF-κB binding site. However, a clear motif is recovered for the E, β and N boxes. We could not detect a significant conservation of nucleotides surrounding the motifs of the boxes (Additional file 3). In line with previous publications, we identified the E box 'logo' as CAGCTG. This consensus sequence binds strongly to daughterless-AS-C heterodimers. Moreover, unlike ASC homodimers, the binding of Ase-Ase homodimers to this site was observed [13]. It is interesting to note that E boxes with the CAGCTG logo are present once in each arthropod species, although, overall, the motif is present in only 5 of the 17 E boxes identified.

### The SOPE of *C. salei CsAHS2 *and *T. castaneum ase *are functional in *D. melanogaster*

**Figure 5:**
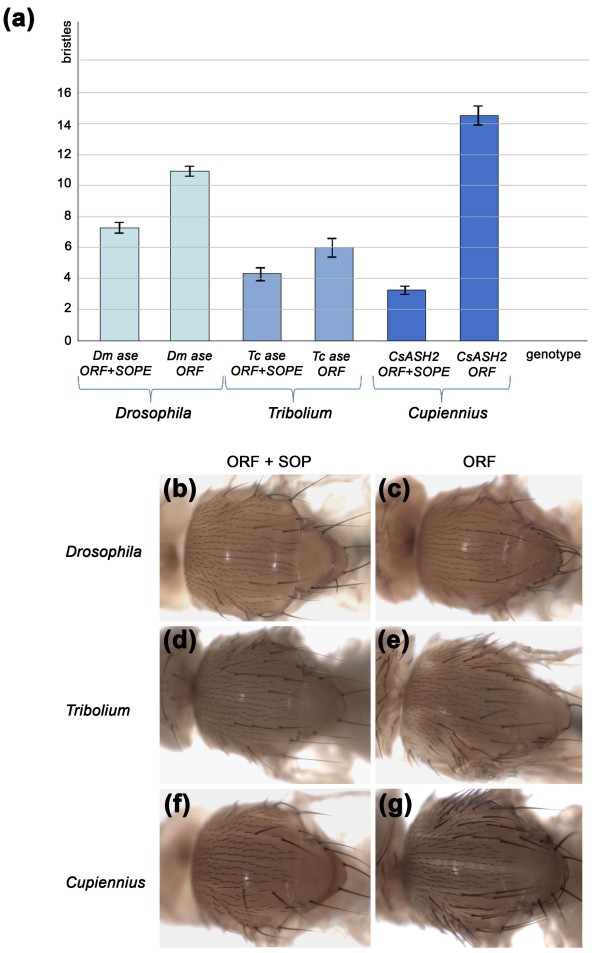
**Comparison of the number of ectopic bristles (macrochaetes) displayed by transgenic flies after ectopic expression**. UAS constructs containing *D. melanogaster ase*, *T. castenum ase *and *C. salei CsASH2 *(ORF alone or the entire transcribed region (ORF+SOPE) of *ase *or *CsASH2*) were each crossed to four different *Gal4 *drivers allowing expression in different parts of the thorax (see Materials and methods). **(A) **The number of ectopic bristles was counted in the respective *Gal4 *expression domains of each of the driver lines. Columns give the number of ectopic bristles and data from all four crosses have been pooled for each UAS construct (UAS-ORF+SOPE or UAS-ORF). The number of ectopic bristles is significantly reduced in flies carrying the UAS-ORF+SOPE constructs (see Additional file 4 for details). The error bars give the standard error of the mean. **(B-G) **Thoraces illustrating the phenotypes obtained: (B, C) *ptc-Gal4 > UAS Dm-ase*; (D, E) *sca-Gal4 > UAS Tc-ase*; (F, G) *MD806-Gal4 > UAS CsASH2*.

The *Dm-ase *SOPE had been shown to display enhancer activity when placed upstream of an hsp70 promoter and a reporter gene [13]. To test its effects when positioned in the UTR, we generated transgenic lines carrying UAS constructs containing either the entire transcribed region (including the UTR sequences) or merely the ORF. Since reporter gene fusion constructs that cover different regions upstream of the ORF only restrict expression to single SOPs if the 560-bp UTR containing the SOPE is present [13], the ORF+SOPE constructs should reduce the number of bristles. Three independent lines of each construct were crossed to four different *Gal4 *lines, each of which drives expression in all or part of the *D. melanogaster *notum. As expected, both transgenes caused the development of ectopic bristles but their number was significantly reduced in the construct containing the entire UTR. Flies expressing the *UAS-Dm-ase *ORF displayed, in total, an average of 10.9 ectopic bristles, compared with 7.3 in flies expressing the *UAS-Dm-ase *ORF+SOPE (Figure 5A-C; Additional file 4). We therefore conclude that the SOPE regulates gene activity from its position in the UTR and that, when transcription is initiated from exogenous UAS sequences, it functions to dampen transcription. This is consistent with its proposed function to restrict proneural gene activity from broad expression domains to single neural progenitors.

To see whether the strong conservation of binding sites in the UTR of other arthropod *ase-*like genes is meaningful, we tested the putative SOPEs of *T. castaneum *and *C. salei *for function in transgenic flies. Transgenic lines were made containing UAS sequences and the entire transcribed regions or just the ORFs of *Tc-ase *and *CsASH2*. Three independent lines of each construct were crossed to the same four *Gal4 *lines as above. The number of ectopic bristles was used to measure activity. Flies expressing the *UAS-Tc-ase *ORF displayed an average of 6.0 ectopic bristles, and those expressing *UAS-Tc-ase *ORF+SOPE an average of 4.4 (Figure 5A, D, E; Additional file 4). Flies expressing the *UAS-CsASH2 *ORF displayed an average of 14.3 ectopic bristles, and those expressing *UAS-CsASH2 *ORF+SOPE an average of 3.4 (Figure 5A, F, G; Additional file 4, misexpression experiment). These data indicate that the SOPEs of *T. castaneum *and *C. salei *function in a similar fashion to that of *D. melanogaster*, consistent with the conservation of binding sites in these sequences.

## Discussion

### Subfunctionalization and divergence of *ASH *and *ase*-like genes

Most new genes are thought to arise through gene duplication because of the need to evolve simultaneously signals for regulation and transcript processing. Our data suggest that gene duplications in individual arthropod lineages have led to the segregation of proneural (*ASH*) and precursor-specific (*ase-*like) functions of a single ancestral gene. We show here that *bona fide ase *genes are present in crustaceans. Phylogenetic analysis was unable to resolve whether the *ase *and *ASH *genes of insects and crustaceans are derived from the duplication of an ancestral gene in the last common ancestor of both groups or rather from independent duplications in the individual lineages. However, the presence of the Ase motif in both insect and crustacean *ase *genes would support a common origin. This is consistent with the Tetraconata hypothesis, which suggests a sister group relationship of insects and crustaceans [32]. We are confident that the myriapod *S. maritima *has a single *ASH *gene. This gene would therefore need to perform both proneural (*ASH*) and precursor-specific (*ase*) functions. This is likely to reflect the ancestral state. Myriapod and chelicerate *ASH *genes group together in our phylogenetic analysis. We think this might simply reflect the retention in both groups of many ancestral homologies. Although the phylogenetic position of myriapods is still under debate, most phylogenies are consistent with the Mandibulata hypothesis, which proposes a sister group relationship of Myriapoda and Tetraconata (insects and crustaceans) [33]. The chelicerate lineage represents a basal branch of the arthropods. An independent duplication in chelicerates resulting in two *ac-sc *orthologues is supported by our phylogenetic analysis. Sequence comparison based on the conserved domains of the insect and crustacean genes does not distinguish a proneural *ASH *and a precursor-specific *ase *gene. However, *CsASH2 *is expressed exclusively in neural precursors and contains an SOPE in the transcript. This, together with its ability to rescue the *ase *mutant phenotype in *D. melanogaster*, strongly suggests that *CsASH2 *carries out an *ase*-like function. Together, the data support the hypothesis of subfunctionalization and gradual divergence of arthropod *ASH *and *ase*-like functions.

In *D. melangaster*, the SOPE has been shown to mediate the process of refining transcription from a field of cells to single, spaced precursors [14]. The ability to restrict their own transcription to subsets of progenitors is the most highly conserved process associated with proneural genes throughout the animal kingdom [1]. Our data suggest that, at least in arthropods, this process is linked to the presence of the SOPE. It has been shown recently that upstream fragments outside of the SOPE do not drive reporter gene expression in single cells. Furthermore, mutations of the E boxes abolish the activity of the SOPE enhancer [13]. Thus, we are confident that the reduction of ectopic bristles in our transgenic flies containing the ORF+SOPE results from the activity of the SOPE enhancer. In view of the high level of conservation of the specific binding sites, it is likely that it requires not only auto-regulation and Notch-mediated lateral inhibition but also an important contribution from NF-κB signaling [14,18]. A unique feature of the *ase-*like genes is the location of the SOPE in the UTR of the transcript. The single *ASH *gene of *S. maritima *has retained the SOPE in the transcript. If *S. maritima *does indeed reflect the ancestral condition, it would indicate that the SOPE was present in the UTR of the ancestral *ASH*/*ase *precursor gene. This would place the origin of this regulatory sequence as far back as the last common ancestor of the Arthropoda, that is, in the Cambrian, 550 million years ago.

### Position of the SOPE and evolution of proneural gene expression

It appears that in both chelicerates and Tetraconata, the SOPE has been retained in the transcript of the *ase-*like gene after duplication. In *D. melanogaster *we know that the *ASH *duplicates are also regulated by a SOPE but that it has been dislocated from the transcription unit. Like those of Diptera, expression of the *ASH *genes of crustaceans and spiders is refined from initially broad domains to neural precursors, suggesting that they too are subject to lateral inhibition and the activity of a SOPE [23,24,34]. Therefore, in these species also, a SOPE might reside amongst regulatory sequences outside the transcription unit of the *ASH *genes.

The fact that the SOPE is found in the UTR of all *ase-*like genes, including the single myriapod orthologue, whether 5' or 3', suggests that this location is important. One possible reason is that it is protected here and is less likely to become separated from the gene since re-arrangement would more often lead to mutations and loss of gene activity. Moreover, if the gene comes under the influence of any other regulatory sequences (outside the transcription unit) the SOPE would still be active. Analysis of the activity of the transgenes whose transcription is initiated by Gal4 > UAS sequences indicates that the presence of the SOPE in the UTR dampens activity. Perhaps the protected location is a failsafe mechanism to ensure refinement of expression to single progenitors. In this context it is interesting to note that we identified a putative SOPE enhancer in the 5' UTR of *senseless*, another gene whose expression becomes restricted to SOPs [29,35] (Additional file 5). Alternatively, the SOPE might have been retained in the UTR because it covers an area that contains additional elements for controlling post-transcriptional regulation such as RNA folding. The predicted secondary structure (using the RNAfold WebServer [36]) of the UTRs shows characteristic structures such as stem-loops and pseudoknots (Additional file 6). However, whether these arrangements exert influence on the regulation of the *ase*-like genes remains to be shown.

Separation of the SOPE from the transcription unit in *ASH *genes presumably occurred during (or after) duplication of the ancestral *ASH*/*ase *precursor gene. In *D. melanogastser*, the *Dm-sc *SOPE is 3 kb upstream of the transcription unit and, furthermore, another *cis-*regulatory element is situated between the SOPE and the transcription start site [14,37]. *Dm-sc *is subject to regulatory input from an array of independently acting enhancer elements, in addition to the SOPE, each of which has to be brought into close proximity to the basal promoter to drive expression in distinct regions [9,38,39]. One consequence of this is that the SOPE is probably only active at certain times. In contrast, the *Dm-ase *SOPE would continuously modulate the rate of transcription after initiation from the basal promoter by virtue of its position in the UTR.

After duplication of the ancestral *ASH*/*ase *gene, the SOPE appears to have been disconnected from the transcript of the duplicate that becomes the proneural *ASH*. This event is likely to have occurred before the divergence of insects and crustaceans. A similar occurrence might have taken place convergently in chelicerates. We suggest that disconnection of the SOPE from the transcript has facilitated the greater complexity of spatial and temporal regulation that underlies the diversity of patterning of the nervous system in arthropods. This could have unfolded during evolution as follows. The common ancestor of the Arthropoda probably had a single *ASH*/*ase*-like gene, similar to that of the extant myriapods. It would have been expressed ubiquitously over the neuro-epithelium and subsequently restricted to single precursors. This ancestral expression pattern can be observed today in Onychophora, the closest relative of the arthropods (B. J. Erkisson and A Stollewerk, unpublished). Transcriptional modulation would have been mediated by the SOPE, located in a protected position in UTR sequences. Gene duplication followed by subfunctionalization resulted in proneural *ASH *and precursor-specific *ase-*like genes independently in Tetraconata and chelicerates. Retention of the SOPE in the transcript of *ase-*like genes ensures its expression in SOPs. Loss of the SOPE from the transcript allowed *ASH *expression to be spatially regulated by other (non-transcribed) *cis*-regulatory sequences. An independent transcriptional regulation would not be effective in the *ase-*like genes because of the presence of the SOPE in the UTR. *Cis-*regulatory elements for spatial expression might have been acquired more recently. Indeed, the most complex regulation of *ASH *genes is seen in cyclorraphous flies, where expression in small clusters of cells at precise positions prefigures the development of large sensory bristles, macrochaetes. Macrochaetes are an evolutionary novelty of higher flies and are found in species-specific patterns. In *D. melanogaster *the patterns rely on an array of independently acting *cis-*regulatory elements [9] that are likely to have arisen in the Cyclorrapha along with the additional duplication events of the ancestral *ASH *gene [25,40].

## Conclusions

An increasing number of publications demonstrate conservation of function of *cis*-regulatory elements without sequence similarity (reviewed by [41]). In vertebrates the functional conservation even spans the evolutionary distance between humans and zebrafish [42]. In invertebrates such functional conservation has only been shown for closely related species that diverged from their common ancestor not longer than 25 to 60 million years ago (for example, [43,44]). Our results demonstrate for the first time the existence of an ancient arthropod regulatory element dating back to the Cambrian (about 500 million years ago). The element shows a conserved function but without sufficient sequence conservation to be detected on the basis of sequence alignment, opening the possibility that other ancient invertebrate regulatory elements remain to be discovered.

## Materials and methods

### *Drosophila *culture and stocks

Flies were maintained on standard cornmeal-agar medium at 18°C and Oregon-R was used as a control. Strains used were: *ase^1 ^*(formerly known as *sc^2 ^*[10]), *toll-8[MD806] Gal4 *[18], *ptc-Gal4*, *sca[537.4] Gal4*, *achaete[SBM] Gal4 *[45], *UAS-sc *(FlyBase [46]). UAS-constructs for ectopic expression of *D. melanogaster *and *T. castaneum ase *and *C. salei CsASH2 *were generated by standard techniques. P-element-mediated transformation was performed by standard techniques.

### Rescue experiment

*ase^1 ^*flies were crossed to *hsp70Gal4 > UAS Dm-ase*, *hsp70Gal4 > UAS Tc-ase *and *hsp70Gal4 > UAS CsASH2 *flies, respectively, and allowed to lay eggs in culture bottles for 3 days. Heat shocks were preformed between 16 hours and 8 hours before puparium formation. Heat shock expression was driven by three 1-hour heat shocks at 37°C, separated by 2 hour intervals at 25°C. Wings were mounted in glycerol and analyzed under a compound microscope (Leica).

### Bristle scoring

Three independent lines were generated for each UAS construct (ORF only and ORF+SOPE) and crossed to four different *Gal4 *lines that activated the constructs in the expression domains of *toll-8*, *patched*, *scabrous *and *ac*. Flies of the appropriate genotype were selected, mounted and the bristles were counted under the dissecting microscope (Leica). Statistical analysis was performed using Microsoft Excel.

### Identification of *ASH *and *ase *genes

The sequenced genomes of *D. pulex *(*Daphnia_pulex *2006-09 JGI) and *S. maritima *(*Strigamia maritima *Genome Project by Baylor College of Medicine, NCBI Project ID 20501) were searched using tblastn with the ASH and Ase proteins of *D. melanogaster*, *T. castaneum *and *C. salei *as queries. Hits with relevant homology to the bHLH domain were further characterized. Three genes were identified in the *D. pulex *genome: an *ASH *homologue (JGI_V11_254034), an *ase *homologue (JGI_V11_254038) and a truncated copy of *ase *(JGI_V11_232740). In the *S. maritima *genome only one *ASH *homologue was identified; a second gene analyzed was too divergent in the basic region of the bHLH domain to be classified as an *ASH *gene.

### Phylogenetic analysis

Phylogenetic trees were constructed using all *ASH *and *ase *genes from the insects *D. melanogaster *and *T. castaneum*, the crustaceans *T. longicaudatus *and *D. pulex*, the chelicerate *C. salei *and the myriapods *G. marginata *and *S. maritima*. Amino acid sequences have been aligned with ClustalW2 [47], manually improved and conserved regions selected with Gblocks (using permissive parameters) [48]. The resulting alignment is only 66 amino acids long and corresponds roughly to the basic region and helixes of the bHLH domain and to the carboxy-terminal domain. Trees have been constructed by maximum likelihood methods and tree topologies compared with the Shinodaira-Hasegawa test as implemented in the Phylip package [49].

## Abbreviations

*AC*: *achaete*; AS-C: Achaete-Scute complex; *ASE*: *asense*; *ASH*: *achaete-scute homologue*; *ATO*: *atonal*; BHLH: basic helix-loop-helix; BP: base pair; NF: nuclear factor; *SC*: *scute*; ORF: open reading frame; SOP: sensory organ precursor; SOPE: sensory organ precursor enhancer; UTR: untranslated region.

## Competing interests

The authors declare that they have no competing interests.

## Authors' contributions

Conceived and designed the experiments: SA, PS, AS. Performed the experiments and analyzed the data: SA, BN, AS. Wrote the manuscript: AS, PS. Contributed to writing and editing: BN.

## Supplementary Material

Additional file 1 ***asense *rescue experiment**. Comparison of the number of stout bristles exhibiting differentiation defects in *ase^1 ^*flies (first column) and *ase^1 ^*flies carrying *hsp70Gal4 > UAS Dm-sc*, *hsp70Gal4 > UAS Dm-ase*, *hsp70Gal4 > UAS Tc-ase *and *hsp70-Gal > UAS-CsASH2 *transgenes Click here for file

Additional file 2 **Alignment of the SOP enhancer elements**. Nucleotide sequence alignments of the individual transcription factor binding sites of *Drosophila*, *Tribolium*, *Daphnia*, *Strigamia *and *Cupiennius*. Click here for file

Additional file 3 **Sequence logos of the SOPE boxes**. Graphic representation of the aligned sequences of the SOPE binding sites of *Drosophila melanogaster*, *Tribolium castaneum*, *Daphnia pulex*, *Strigamia maritima *and *Cupiennius salei*. Click here for file

Additional file 4 **Misexpression experiment**. Comparison of the number of ectopic bristles in flies carrying the *UAS-ase/ASH2 *ORF only and ORF+SOPE constructs. Click here for file

Additional file 5 **SOP enhancer in the *senseless *5' UTR**. Arrangement of the SOPE boxes in the 5' UTR of the senseless transcript. Click here for file

Additional file 6 **Secondary structure of the *ase*-like UTRs**. Graphic representation of the secondary structure of the UTRs of the *Drosophila melanogaster*, *Tribolium castaneum *and *Daphnia pulex asense *genes and the UTRs of *Strigamia maritima ASH *and *Cupiennius salei CsASH2*. Click here for file
