# Supplementary material for: An arthropod cis-regulatory element functioning in sensory organ precursor development dates back to the Cambrian
Source: BMC Biol. 2010 Sep 24;8:127. doi: 10.1186/1741-7007-8-127 (PMC2958161; doi:10.1186/1741-7007-8-127)
Supplement: Additional file 1 — asense rescue experiment. Comparison of the number of stout bristles exhibiting differentiation defects in ase1 flies (first column) and ase1 flies carrying hsp70Gal4 > UAS Dm-sc, hsp70Gal4 > UAS Dm-ase, hsp70Gal4 > UAS Tc-ase and hsp70-Gal > UAS-CsASH2 transgenes [file 1741-7007-8-127-S1.pdf]

Additional figure 1: *Asense* rescue experiment. Comparison of the number of stout bristles exhibiting differentiation defects in *ase*^{1} flies (first column) and *ase*^{1} flies carrying *hsp70Gal4>UAS Dm-sc*, *hsp70Gal4>UAS Dm-ase*, *hsp70Gal4>UAS Tc-ase* and *hsp70-Gal>UAS-CsASH2* transgenes (251 anterior wing margins were analyzed). Note that the *ase*^{1} phenotype is enhanced in the *hsp70-Gal4>UAS-Dm-sc* line, that was used as a negative control. The small numbers give the standard error of the mean.

	ase^{1}	*Dm scute*	*Dm asense*	*Tc asense*	*CsASH2*
affected bristles	9.72<br>0.44	13.56<br>0.89	2.52<br>0.12	4.86<br>0.39	3.84<br>0.33
