# Supplementary material for: An arthropod cis-regulatory element functioning in sensory organ precursor development dates back to the Cambrian
Source: BMC Biol. 2010 Sep 24;8:127. doi: 10.1186/1741-7007-8-127 (PMC2958161; doi:10.1186/1741-7007-8-127)
Supplement: Additional file 2 — Alignment of the SOP enhancer elements. Nucleotide sequence alignments of the individual transcription factor binding sites of Drosophila, Tribolium, Daphnia, Strigamia and Cupiennius. [file 1741-7007-8-127-S2.pdf]

Sequence comparison of α-boxes

| | |
|---|---|
| *Dm scute* α1 | ---TAAGCCCGTAT-- |
| *Dm scute* α2 | ---GCAACCCCTA--- |
| *Dm scute* α3 | ----GCTCCCCTATTT |
| *CsASH2* α1 | ----TCGCCCCT---- |
| *Sm ASH* α1 | ----TCTCCCCTGG-- |
| *Sm ASH* α2 | ----CCACCCCT---- |
| *Sm ASH* α3 | TGGGAGTCCCC----- |
| *Dm asense* α1 | TTTTCATCCCCTAG-- |
| *Dm asense* α2 | TGGGTGTCCCTT---- |
| *Tc asense* α1 | ---GATTCCCCT---- |
| *Tc asense* α2 | ---GAATCCCC----- |
| *Dp asense* α1 | ----ATTCCCCT---- |

Sequence comparison of β-boxes

| | |
|---|---|
| *Dm scute* β1 | GTTATTTTT- |
| *Dm scute* β2 | GTTCATTTT- |
| *Dm scute* β3 | --GCATTTT- |
| *CsASH2* β1 | --TAGTTTTT |
| *Sm ASH* β1 | GTTTTTTTGT |
| *Sm ASH* β2 | GTTTATTT-- |
| *Sm ASH* β3 | GTCAGTTTT- |
| *Dm asense* β1 | GTTAGTTTT- |
| *Tc asense* β1 | GTTACTTTT- |
| *Tc asense* β2 | -TTATTTTTT |
| *Tc asense* β3 | -TTCGTTTTT |
| *Tc asense* β4 | -TTATTTTTT |
| *Tc asense* β5 | -TTATTTTTT |
| *Dp asense* β2 | GTTCTTTCT- |
| *Dp asense* β1 | GCTATTTTC- |

Sequence comparison of E-boxes

| | |
|---|---|
| *Dm scute* E1 | CACCTG |
| *Dm scute* E2 | CAGCTG |
| *Dm scute* E3 | CAAATG |
| *CsASH2* E1 | CATCTG |
| *CsASH2* E2 | CAGCTG |
| *CsASH2* E3 | CATATG |
| *Sm ASH* E1 | CATATG |
| *Sm ASH* E2 | CACGTG |
| *Sm ASH* E3 | CAGTTG |
| *Sm ASH* E4 | CACCTG |
| *Dm asense* E1 | CAGCTG |
| *Dm asense* E2 | CAAATG |
| *Dm asense* E3 | CAGATG |
| *Dm asense* E4 | CAGGTG |
| *Tc asense* E1 | CAGCTG |
| *Tc asense* E2 | CATGTG |
| *Dp asense* E1 | CAGCTG |

Sequence comparison of N-boxes

| | |
|---|---|
| *Dm scute* N-box | CACGCG |
| *Sm ASH* N-box | CACGCG |
| *Dm asense* N-box | CACGCG |
| *Tc asense* N-box | CACGCG |
| *Dp asense* N-box | CACGAG |

**Additional file 2:** Alignment of the SOP enhancer elements of *Drosophila melanogaster*, *Tribolium castaneum*, *Daphnia pulex*, *Strigamia maritima* and *Cupiennius salei*. Boxes in *Drosophila* enhancers have been described previously (Culi and Modolell 1998). In all other species the individual binding sites were identified by manual screening based on consensus sequences (E-box: CANNTG; α-box: GGRRADYCCCY; β-box: GTTMDTTTT; N-box: CACGMG).
