# Supplementary material for: An arthropod cis-regulatory element functioning in sensory organ precursor development dates back to the Cambrian
Source: BMC Biol. 2010 Sep 24;8:127. doi: 10.1186/1741-7007-8-127 (PMC2958161; doi:10.1186/1741-7007-8-127)
Supplement: Additional file 3 — Sequence logos of the SOPE boxes. Graphic representation of the aligned sequences of the SOPE binding sites of Drosophila melanogaster, Tribolium castaneum, Daphnia pulex, Strigamia maritima and Cupiennius salei. [file 1741-7007-8-127-S3.pdf]

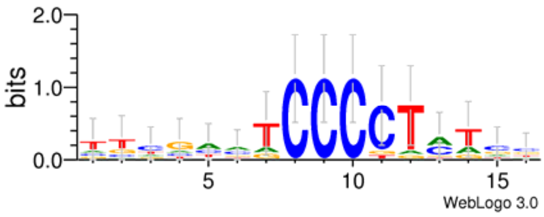

α-box

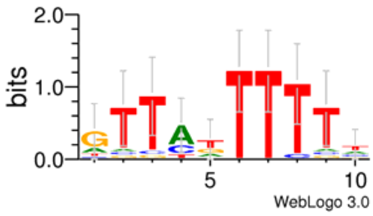

β-box

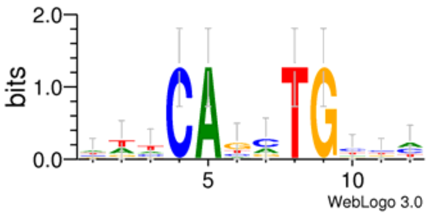

E-box (plus 3 nt on each side)

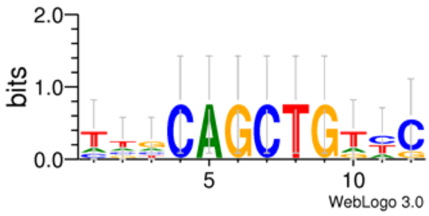

CAGCTG-E-boxes (plus 3 nt on each side)

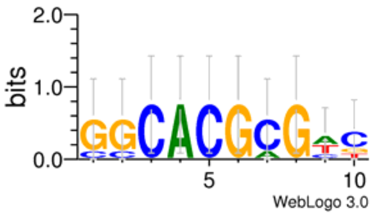

N-box (plus 2 nt on each side)

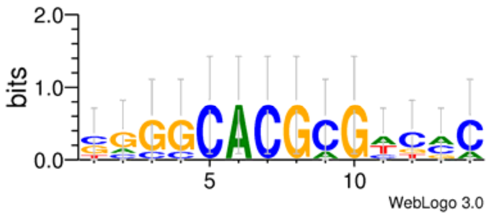

N-box (plus 4 nt on each side)

Additional file 3: Sequence logos of the SOPE boxes. Graphic representation of the aligned sequences of the SOPE binding sites of *Drosophila melanogaster*, *Tribolium castaneum*, *Daphnia pulex*, *Strigamia maritima* and *Cupiennius salei*. Each logo consists of stacks of symbols, one stack for each position in the sequence. The overall height of the stack indicates the sequence conservation at that position, while the height of symbols within the stack indicates the relative frequency of each nucleic acid at that position. The consensus sequence of the extended N-box (2 nt at each side) show the high-affinity binding site for Hairy/Enhancer of Split repressor proteins (Rebeiz et al., 2005; Dev Biol 281:299-308). See text for further details.
