# Supplementary material for: An arthropod cis-regulatory element functioning in sensory organ precursor development dates back to the Cambrian
Source: BMC Biol. 2010 Sep 24;8:127. doi: 10.1186/1741-7007-8-127 (PMC2958161; doi:10.1186/1741-7007-8-127)
Supplement: Additional file 4 — Misexpression experiment. Comparison of the number of ectopic bristles in flies carrying the UAS-ase/ASH2 ORF only and ORF+SOPE constructs. [file 1741-7007-8-127-S4.pdf]

Additional file 4: Misexpression experiment. Comparison of the number of ectopic bristles in flies carrying the *UAS-ase/ASH2* ORF only and ORF+SOPE constructs. Three independent lines were generated for each UAS construct and four different Gal4 lines were used that activated the constructs in the expression domains of *toll-8*, *patched*, *scabrous* and *achaete*. The small numbers give the standard error of the mean. See Fig 5 for additional details.

	Dm asense ORF +SOPE	*Dm asense* ORF	*Tc asense* ORF +SOPE	*Tc asense* ORF	*Cs CsASH2* ORF +SOPE	*Cs CsASH2* ORF
ectopic bristles	7.34 0.35	10.91 0.33	4.35 0.39	5.97 0.59	3.36 0.29	14.34 0.58
