# Supplementary material for: An arthropod cis-regulatory element functioning in sensory organ precursor development dates back to the Cambrian
Source: BMC Biol. 2010 Sep 24;8:127. doi: 10.1186/1741-7007-8-127 (PMC2958161; doi:10.1186/1741-7007-8-127)
Supplement: Additional file 5 — SOP enhancer in the senseless 5' UTR. Arrangement of the SOPE boxes in the 5' UTR of the senseless transcript. [file 1741-7007-8-127-S5.pdf]

AACGATGATAGATGTGTGAGCGGCATACGGTGAATCCGTTACAAAGCGGCAGGGGATGAGCACGCGCCCTCAGTT
CTCCAGATAAACAATATGTATGGGAACGCCGCCAAGCGCAACGATGCCACCAACTTGACTTGGCCAGCCCTCATC
GCGGTCGAAATGGTAGAAGCCAGAGTTCCCCCATTTTGGCCCGCTTTTAGGCCGCGTGCCTCGTACCGCTTCGCT
AGATCGGCGGCACCCGTGGCGGACACGAGTGCGATGGAATTTCGGTGTCGCCTCAACTCTGCACATGTCCTGCCA
TGGAAATTGCGCCAAAAACTAGGGGATGGGAACTAAAATTGCTGACAAGGAAAAGGATCGTTAAATGTGGAAGTG
TAAGTGTGCGCCCGCCACATTTTCTTGGCTCGTTTTCTAGGCAGGTGTGGCCCGCGCCAAGGATAGATAAACTGG
AATTGAGGCGGCCAAGACGAGGTGGCCAAGCGTGGCGAGCTACCAACTCAGGAGGAGGACCCCGAACTCTCATCA
AGGATAAGCTCGGAAAAAGGTGCATCGAGCGGTAGAGGGAGAAAGTGGCAGGTGCTCAGTCGTCCTATCTGAGTG
GTTTAATAGCTACGATCCAACGAGAAGATGGGGACTTTATGGCTTAGAGTTTGGTATTGGTTTTAAATTGACTGA
CAAAAGAATTCAGTCTTTTAATTTTATTGAAATGCTTTAGGAAAATACGACGAACTGCAAAGTTTGCATTGAGAA
ATTTAACAAAAACTTTGTTGAAAGTTAATGTTCTGAAACCAAACCAAACTATTTGTTAAAACGGTTTTAAATGGG
AAATCTGATATTTTGATAGCTATTAAATAAAATCTGTCCTAATCTTATGTATTTAAATGTAACATTATTATAATC
AAATATGTCATTTTGGTAAGGTAGATTTTATATAGAAATATAACAAAAAAAATTCTACAATAATTTAAATAAAGT
TTATATATTTGTGCATTGGTTGTGAAGTAGGTTTACGTCAATCCTCTAAAATGTAATAACACTTTTTATGGATGA
AGTCTAAAGCTTAAAGATTAAATTCACGATGTTGCTTTAAACTATTTACCCTTATTGGGAGAAGCGCAAATAAAA
ATGAATTTAAACTTAATTTCTCCTATATTTTACTCGCAATGTATTTTTAATAATTAATACAAAAATTAATTTTCA
TCACATTCAAAATTAGGAACCCATTTGTAATCCTTGTCAAATCTAAAGGTAATCTAAACAGTATCTACAAACATG
GAGAGTTGCCACGACAGATGTTAGATCCTTGGATTCATCCACCTAAATATTTGTATGCTACCCCGCCCCATCCCG
CCCTCGTCCTCTCCCCCCTTCCACCGATCAGCAGTGAAATCCTCTGCCGATTGCTGGCCCTCTCTCGCTTCTTCT
CCCTTGGCCAAAGTACCGATCCCGATATTCCTGAGCCAACAACAGTTGGTGGCAGCAACAGAAAGAGCAAAGGCA
ATTGTAACTGTTACAGGGCAGCGGGAAAAGGATAACAAACGCCCGCCAAACTTTAAGTGCATACCAATTAGTAGG
GAAACGCGTGCTGGAAAACCAGTAGTAGATGGGCCAAGAGGATACGAACGCAGGGGGTTGGGGTATCGAGATCGC
CAGGGGACTCCTAACTGTGCGTCTTGACCCATACAACATGGAACACAGGACTGCAACTGCCGCCAACTGCTGCCA
AAGGTGAAAAACCAGAGTTGGCAACACGAACACCAACACCAACAATACATTGCAGGGCTCTCGAGAGCCACCACC
AAAAGAGTCAACACCAATACACCGAGGGCTCGTTTTGATTCGGCTTGCTGCCGCACTCACACAGTGCCACAAGTA
ACCACAGTCGCACACAAAAATAAAAAAAAGAGAGCTCGATTTGTACCTTTTTTTCGGGCCAAGTCGCTTCACCGT
TGGCTCAATTTAGAACTGAGCCATCGACCAAAGATTCAGTCAGGTGCGAAGCACCGAGCGATACGGACGTGTTCG
TCTCCAGTGAGTCAAGATAAAACCGCACTGAATAAGACCCATCGAAGATCCGTTCGAATCCAAGATCACAACGCC
AAGAAAGTGAGATTTGTGCAGTGAACAGTATTGAGAAAAATCACAATCCAAAGACTGTTAAAGCAAATACGTAAA
TCAGATCCAGTGAGATTCCAAAAAGTAAAGATCCAGAATCAAAATGAATCACCTATCGCCGCCGCCATCGCCGCA
CTCCCAGCAGCCGAGTCCCGCGGGATTGGGCTGCCATGGAGCAGCTCTGGACAAGCAGTGGATGCAGCGGGCATC
CGCCTTCAACACTGTGATAGCCTCCGCCGCAGCACAGAAACTCAATGGTCGAGGTGAGTTCCCGATCTGGATTGA
CAGAAAAATATATGGGGCAGTAAAGATAGCAAAAAGATTAGCTTATAGCTTTTTAATCGTTTCGAATTGTATTTA
AAAGATCACTCGATTTAAAATCAAAATGTTAACACTTTCAACAGATCAGAAAAATTATGTTAACTTTACTGTAGT

Additional file 5: SOP enhancer in the *senseless* 5’ UTR. The 5’ UTR contains 6 E-boxes (yellow), 4 α-boxes (pink), 3 β-boxes (green), 1 N-box (blue), translation start site (red).
