# Supplementary material for: An arthropod cis-regulatory element functioning in sensory organ precursor development dates back to the Cambrian
Source: BMC Biol. 2010 Sep 24;8:127. doi: 10.1186/1741-7007-8-127 (PMC2958161; doi:10.1186/1741-7007-8-127)
Supplement: Additional file 6 — Secondary structure of the ase-like UTRs. Graphic representation of the secondary structure of the UTRs of the Drosophila melanogaster, Tribolium castaneum and Daphnia pulex asense genes and the UTRs of Strigamia maritima ASH and Cupiennius salei CsASH2. [file 1741-7007-8-127-S6.pdf]

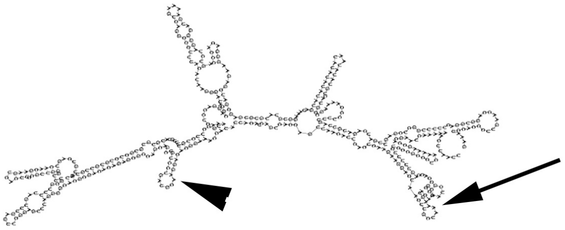

Drosophila melanogaster

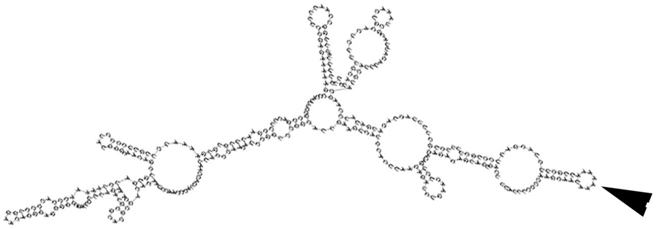

Tribolium castaneum

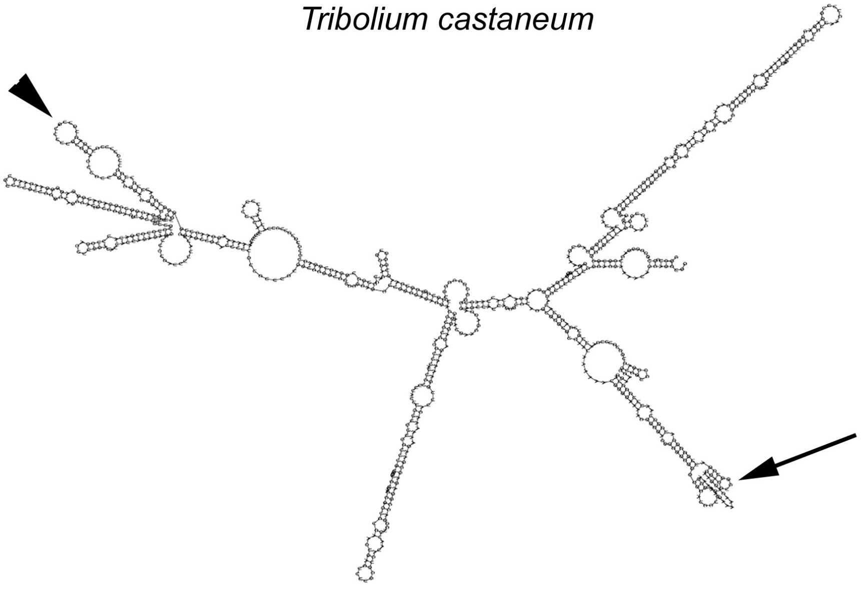

Daphnia pulex

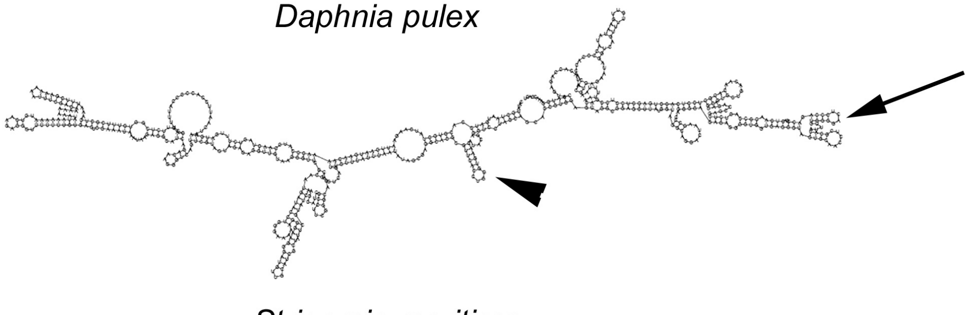

Strigamia maritima

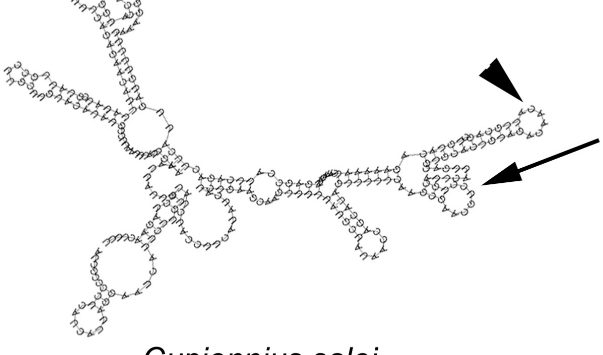

Cupiennius salei

Additional file 6: Secondary structure of the *ase*-like UTRs. Graphic representation of the secondary structure of the UTRs of the *Drosophila melanogaster*, *Tribolium castaneum* and *Daphnia pulex asense* genes and the UTRs of *Strigamia maritima ASH* and *Cupiennius salei CsASH2*. The predicted RNA folding (http://rna.tbi.univie.ac.at/cgi-bin/RNAfold.cgi) shows stem-loops (arrowheads) and pseudoknots (arrows).
